# The impact of a physical exercise program on quality of life, fatigue, physical performance, and level of physical activity in patients with cancer

**DOI:** 10.1590/1806-9282.2024S120

**Published:** 2024-06-07

**Authors:** Fabiana Reis, Ana Carolina Caporali Pereira, Elisângela Pinto Marinho de Almeida, Rebeca Boltes Cecatto, Christina May Moran de Brito

**Affiliations:** 1 Universidade de São Paulo, Cancer Institute of the State of Sao Paulo, Hospital das Clínicas, School of Medicine, Rehabilitation Service – Sao Paulo (SP), Brazil.; 2 Universidade Nove de Julho, Post Graduate Program in Biophotonics Medicine – Sao Paulo (SP), Brazil.; 3 Hospital Sírio-Libanês, Rehabilitation Service – Sao Paulo (SP), Brazil.

**Keywords:** Neoplasms, Rehabilitation, Physical activity, Fatigue, Quality of life

## Abstract

**OBJECTIVE:**

Increasing evidence suggests that exercise programs are of great value in the rehabilitation and survivorship of patients with cancer. However, challenges remain regarding maintaining patients more physically active. This study aimed to evaluate the impact of a supervised exercise program on quality of life, fatigue, physical performance, and levels of physical activity of patients with cancer.

**METHODS:**

An observational longitudinal study, with a 1-year prospective follow-up, was developed.

**SETTING:**

This is a university-based outpatient rehabilitation program in a high-complexity cancer care center in Sao Paulo.

**RESULTS:**

After the program, patients showed a significant gain in quality of life (p<0.0001), physical performance (p<0.0001), and improvement in fatigue (p<0.0001). After 12 months, 81.1% of the patients remained active, and only 4.5% declared themselves to be sedentary.

**CONCLUSION:**

The results of this study confirm that exercise programs are an important tool in the rehabilitation of patients with cancer and that an initial supervised exercise program, in combination with follow-ups, can help increase the levels of physical activity of this population.

**CLINICAL REHABILITATION IMPACT:**

This study provides additional information on the outcomes that are expected with the provision of a supervised physical exercise program in the rehabilitation care of patients with cancer and that additional follow-ups could further benefit this population.

## INTRODUCTION

Cancer rehabilitation should be integrated throughout the oncology care continuum to maintain or restore function, reduce symptom burden, maximize independence, and improve quality of life in this medically complex population. In many countries around the world, cancer is one of the most frequent causes of morbidity and mortality. According to the World Health Organization, cancer is the second leading cause of death in the world and was responsible for 9.96 million deaths in 2020. The estimated number of new cases (incidence) of different types of cancers, worldwide, is around 10 million a year^1^.

Similar to the incidence of cancer worldwide, the population of cancer survivors continues to grow. Improvements in care are responsible for longer life expectancy and better survival. However, the disease itself and its treatment can have both physical and psychological negative effects, including muscle atrophy, altered body weight, pain, depression, fatigue, an overall reduction in quality of life, bone loss, and functional decline^2^. For this reason, physical activity has been increasingly recognized as an important tool for the recovery and rehabilitation of individuals with cancer^2–6^. There is abundant evidence on the benefits of rehabilitation interventions and physical exercise for patients with cancer, with significant impact on functionality, mobility, physical capacity, mood, self-image, and management of lymphedema^2–7^.

For physical activity to function as a determinant of health promotion and the prevention and reduction of risks associated with diseases in patients with cancer, it must be performed regularly and consistently^8^. The last Physical Activity Guidelines for Americans and many guidelines published earlier recommend at least 150 min per week of aerobic activity of moderate intensity or 75 min of vigorous activity for good health, which was also recommended in the last World Health Organization guidelines on physical activity and sedentary behavior^9,10^.

Exercise, particularly supervised exercise, effectively improves quality of life and physical function in patients with cancer across different demographic and clinical characteristics during and following treatment^4^. However, cancer survivors tend to decrease their level of physical activity following diagnosis, and most never return to their pre-diagnosis levels after treatment^11^.

Thus, measures of physical activity are important because they can provide indicators to assess the health situation of patients with cancer, enabling the planning of interventions that can both promote physical activity and reduce exposure to other risk factors for sedentary behaviors. The objective of this study was to evaluate the impact of a physical exercise program on the quality of life, physical performance, and fatigue levels of patients with cancer and to verify the continuity of their post-discharge levels of physical activity.

## METHODS

**Figure 1:**
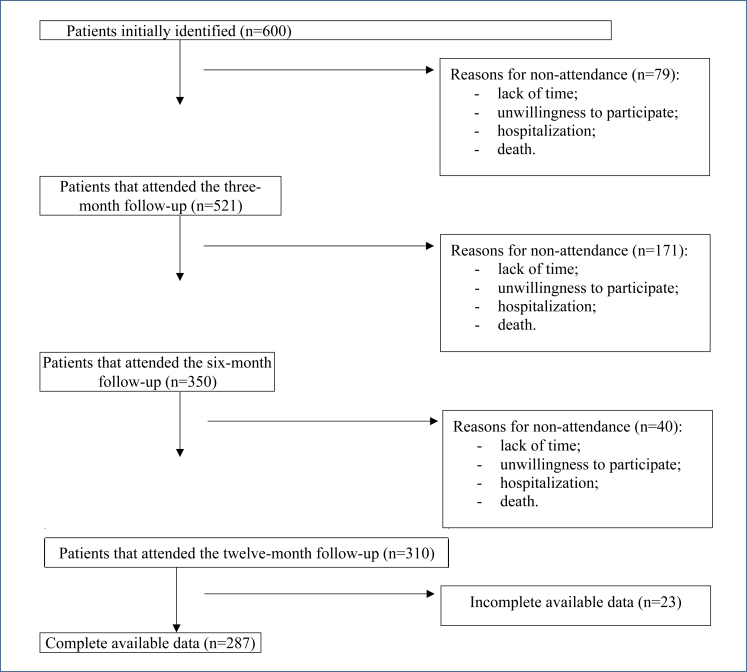
Participants’ follow-up attendance throughout the study.

A prospective observational longitudinal study was developed, involving cancer outpatients of a university-based high-complexity cancer care center in Sao Paulo. A total of 600 adult patients with cancer who participated in an outpatient physical rehabilitation program at the Cancer Institute of the State of São Paulo were recruited for this study. After completing the 3-month exercise program and education, the patients were referred to three post-discharge meetings: 521 patients attended the first meeting (3 months after discharge), 350 patients attended the second meeting (6 months after discharge), and 310 patients attended the third meeting (12 months after discharge). The total data available for analysis involved 287 patients (Figure 1). All the procedures of this study were approved by the Research Ethics Committee of the University of Sao Paulo School of Medicine (approval no: 1.306.807).

Inclusion criteria: the study included patients with cancer who participated in an outpatient physical rehabilitation program and who completed the physical exercise program consisting of 1-h sessions, twice per week, for 3 consecutive months. Exclusion criteria: patients who did not complete the physical fitness program or had an adherence of less than 80% were excluded.

The structured supervised exercise program in this study involved two 1-h sessions per week, for 3 months, and consisted of aerobic, resistance, and flexibility exercises. Patients received specialized medical evaluation by a physiatrist and were referred to a supervised exercise program taking into consideration their clinical and functional conditions and rehabilitation needs. The aerobic exercises were performed on a treadmill, stationary bike, and/or step machine for up to 25 min. Resistance exercises included exercises for major muscle groups (chest, back, arms, and legs), which varied according to the patient's limitations and condition, with a maximum of five muscle groups being exercised per session using weights, dumbbells, and pulleys for up to 25 min. Weekly progression was considered, according to performance and tolerance. The flexibility exercises were performed at the end of each session for up to 10 min, and each position was maintained for 30 s and repeated three times. During all exercises, the patients were monitored for heart rate, blood pressure, scale of perceived exertion (Borg), and oxygen saturation. Patients also received education on the importance of a regular amount of 150 min of moderate to vigorous exercise every week and a booklet with additional information on the benefits of physical exercise and the different types of exercises that should be practiced.

The patients participated in two assessments: one prior to beginning the exercise program and the other at the end of the 3-month program. In both assessments, the patients completed the Revised Piper Fatigue Scale (PFS-R). The PFS-R has 22 items, each rated on a 0–10 numeric scale, and four subscales that assess four dimensions of fatigue: sensory, affective, cognitive-emotional, and behavioral. Both the total score and each subscale score range from 0 to 10, with fatigue scores being categorized as mild (1–3), moderate (4–6), or severe (7–10 points)^12^. Quality of life was assessed using the Short Form-36 Health Survey (SF-36) questionnaire, which is a multidimensional questionnaire consisting of 36 items sorted into eight domains^13^. The 6-min walk test (6MWT) was also performed to evaluate the global and integrated responses of all the systems involved in exercise, including the pulmonary and cardiovascular systems, systemic circulation, peripheral circulation, blood, neuromuscular units, and muscle metabolism. The self-paced 6MWT assesses the patient's submaximal level of physical capacity^14^. In addition, at 3, 6, and 12 months after discharge from the physical exercise program, the short version of the International Physical Activity Questionnaire (IPAQ) was given to patients. The short version of the IPAQ consists of seven open-ended questions, and the resulting information enables estimating the time spent per week in different dimensions of physical activity (e.g., moderate and vigorous walking and physical exertion) and inactivity (e.g., a sitting position)^15^.

The main outcome was the level of physical activity (IPAQ) and the secondary outcomes were fatigue (PFS-R), quality of life (SF-36), and physical capacity (6MWT). Convenience sampling was adopted. Regarding statistical analysis, the normality of the data was tested using the Shapiro-Wilk test, and the Wilcoxon test was used to compare assessments before and after treatment. The chi-square test was used to analyze the level of physical activity. The alpha level was set at p<0.05.

## RESULTS

The mean age of the participants in this study was 58 years, 70% of participants were women, and breast cancer was the most prevalent diagnosis, comprising almost 60% of the cases (Table 1). Patients were either in the active treatment phase or up to 5 years after cancer treatment. After the 3-month program, the patients showed significant differences in their levels of cancer-related fatigue (p<0.0001), quality of life (p<0.0001), and distance walked in the walking test (p<0.0001) (Table 2). We only considered the data of the patients who had at least 80% adherence to the supervised program and of the 287 patients who were successfully followed for 12 months.

**Table 1:** Medical and demographical data.

Patients		n=287	%
Age in years (mean, range, and standard deviation—SD)		58 (21–89; SD 11.7)	
Gender			
	Men	86	30
	Women	201	70
Diagnosis			
	Breast cancer	172	59.9
	Head and neck cancer	30	10.5
	Hematological cancer	18	6.3
	Other tumors	67	23.3

**Table 2:** Values of the Revised Piper Fatigue Scale, Short Form-36 Health Survey (SF-36) questionnaire, and the 6-min walk test.

Variable		Pre	Post	p-value
Fatigue Scale		4.6±2.2	1.8±2.1	0.0001
SF-36		464±157	573±144	0.0001
6-min walk test				
	(Distance in meters)	452±95	523±93	0.0001

Data are mean±standard deviation. Statistically significant p<0.05.

**Figure 2:**
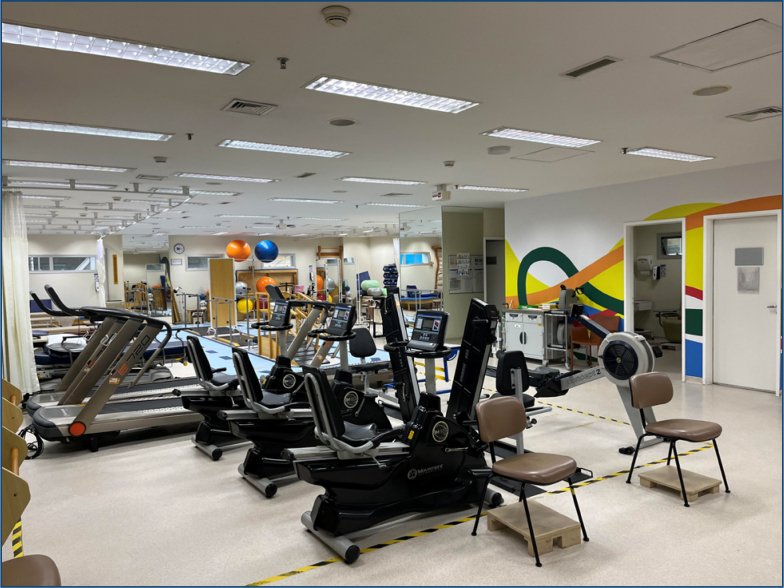
Therapeutic gymnasium of the Cancer Institute of the State of Sao Paulo Rehabilitation Center.

There was no significant difference in the IPAQ questionnaires between the third and sixth months (p=0.8472), the third and twelfth months (p=0.9806), and the sixth and twelfth months (p=0.8491). At 3 months post-discharge, 7.6% of participants were very active, 72.8% were active, 14.9% were irregularly active, and 4.5% were sedentary. At 6 months post-discharge, 7.3% were very active, 74.5% were active, 14.9% were irregularly active, and 3.1% were sedentary. At 12 months post-discharge, 6.9% were very active, 74.2% were active, 14.2% were irregularly active, and 4.5% were sedentary (Table 3). Patients were all sedentary when they started the supervised exercise program. The application of the questionnaires was done by the team of physical educators of the Cancer Institute of the State of Sao Paulo Rehabilitation Center (Figure 2), and the analysis was done by the main researcher.

**Table 3:** Classification of the level of physical activity.

Physical activity status	3 months		12 months		x^2^	p
	n	%	n	%		
Very active	22	7.6	20	6.9		
Active	209	72.8	213	74.2		
Irregularly active	43	14.9	41	14.2		
Sedentary	13	4.5	13	4.5	0.1808	0.9806

IPAQ: International Physical Activity Questionnaire. Comparing months 3 and 12. Values are expressed as absolute numbers and percentages. X^2^ chi-square test. Statistically significant p<0.05.

## DISCUSSION

This study evaluated the impact of a physical exercise program on quality of life, physical performance, fatigue, and adherence to a more active lifestyle in patients with cancer. The results showed statistically significant benefits in most of these aspects. Some researchers claim that the effects of a 12-week exercise program may persist for up to 3 months after the intervention, resulting in a substantial improvement in muscle strength and a decrease in abdominal adipose tissue^16^. Another study showed that 3 months in a physical exercise program can improve cardiovascular capacity, fatigue, and depression symptoms in patients with breast cancer. That study also stated that additional benefits are possible if the exercise is maintained for 6 months^17^.

The literature suggests that although there are some specific risks for patients with cancer that must be considered, physical exercise is generally safe during and after cancer treatment^4,5^. This study corroborates the authors above, showing improvement in the quality of life, a decrease in the oncologic fatigue levels, and improvement in the physical performance of oncology patients. Comparing the results of the final evaluation with those of the initial evaluation clearly demonstrates the importance of a program of exercise in cancer rehabilitation and shows no adverse effects in this studied group. In a systematic review and meta-analysis of 48 randomized clinical trials (3,632 patients), it was shown that aerobic exercise was associated with an increase in cardiorespiratory fitness suggesting that patients with cancer maintain the ability to adapt to the exercise stimulus and that exercise can be effectively combined with other cancer therapies^18^. Similarly, a meta-analysis of 11 randomized controlled trials of resistance training showed that cancer survivors retain the ability to gain muscle strength, increase their lean body mass, and lose body fat in response to this type of exercise while undergoing treatment or long-term follow-up for breast, prostate, or head and neck cancer. It is of clinical relevance to note that no deleterious effects of the exercise program were noted^19^.

Most of the studies in exercise oncology involve patients in the post-adjuvant therapy setting, and the vast majority of studies were conducted in women with breast cancer. The majority of patients in our study also presented breast cancer. In our service, patients with breast cancer are the main group that is referred. The novel aspect of our study was to show the impact of additional follow-ups by specialized physical educators on patients’ behaviors in regard to their level of physical activity. Literature states that the adoption of regular practice of physical exercise after initial supervised programs remains a great challenge^6,11,20^.

In relation to IPAQ data, we must consider that it is a questionnaire that measures different dimensions of physical activity, not just physical exercise. However, for some authors, the limitations of IPAQ include its size, low follow-up adherence, and difficulties completing the questionnaire. These difficulties may be of even greater magnitude for patients with cancer suffering from treatment-related illnesses and side effects, such as fatigue, loss of interest, and cognitive difficulties. In fact, we observed low adherence in response to IPAQ during the 12-month follow-up period, with 87% responding to the IPAQ after 3 months of discharge, 58% responding to IPAQ 6 months after discharge, and only 51% responding to the IPAQ 12 months post-discharge. This might have influenced the results, overestimating the positive effect of the initial supervised program, as the patients exhibiting greater adherence to the follow-up program were probably those more likely to follow orientations and be active. This study showed that after 3, 6, and 12 months post-discharge, a little more than 80% of the patients remained active, and less than 5% declared themselves to be sedentary. Another limitation of our study is that, ideally, physical activity levels should be obtained with more objective measurement systems, such as accelerometers and pedometers, which were not available in this study. Moreover, we opted to consider solely the data regarding the 287 that were successfully followed for 12 months and that had good adherence to the supervised program, which resulted in a selection bias. On the contrary, it also points to the possibility that those with better adherence to the supervised program might probably present higher chances of increasing their physical activity levels in the long run.

In general, our study indicates that a supervised exercise program can encourage the continuity of post-discharge levels of physical activity in patients with cancer, but additional studies are necessary. A recent study with 392 cancer outpatients evidenced that although the great majority of patients (93%) were insufficiently active, 80% declared an interest in exercise programs^21^. In our study, most of the patients declared themselves to be sedentary when starting the rehabilitation program, but our meetings showed that less than 5% of our patients declared themselves to be sedentary during the follow-up period. It is estimated that one-third of adults in the world population do not meet the minimum recommendations for physical activity^22^. Moreover, it is estimated that between only 17 and 58% of cancer survivors adhere to physical activity guidelines^23,24^.

## CONCLUSION

The findings of this study suggest that exercise programs are an important tool in the rehabilitation of patients with cancer and that an initial supervised exercise program, in combination with follow-ups, might contribute to increasing the level of physical activity of some individuals. This study provides additional information on the outcomes that are expected with the provision of a supervised physical exercise program in the rehabilitation care of patients with cancer and that additional follow-ups could further benefit this population.
